# WT1 in Adipose Tissue: From Development to Adult Physiology

**DOI:** 10.3389/fcell.2022.854120

**Published:** 2022-03-16

**Authors:** Karin M. Kirschner, Holger Scholz

**Affiliations:** Charité ‐ Universitätsmedizin Berlin, corporate member of Freie Universität Berlin and Humboldt-Universität zu Berlin, Berlin, Germany

**Keywords:** white adipocyte, brown adipocyte differentiation, thermogenesis, uncoupling protein (UCP), WAT browning

## Abstract

Much of the fascination of the Wilms tumor protein (WT1) emanates from its unique roles in development and disease. Ubiquitous *Wt1* deletion in adult mice causes multiple organ failure including a reduction of body fat. WT1 is expressed in fat cell progenitors in visceral white adipose tissue (WAT) but detected neither in energy storing subcutaneous WAT nor in heat producing brown adipose tissue (BAT). Our recent findings indicate that WT1 represses thermogenic genes and maintains the white adipose identity of visceral fat. *Wt1* heterozygosity in mice is associated with molecular and morphological signs of browning including elevated levels of uncoupling protein 1 (UCP1) in epididymal WAT. Compared to their wild-type littermates, *Wt1* heterozygous mice exhibit significantly improved whole-body glucose tolerance and alleviated hepatic steatosis under high-fat diet. Partial protection of heterozygous *Wt1* knockout mice against metabolic dysfunction is presumably related to browning of their epididymal WAT. In the light of recent advancements, this article reviews the role of WT1 in the development of visceral WAT and its supposed function as a regulator of white adipose identity.

## Introduction

Since the initial discovery that mice with homozygous disruption of the Wilms tumor gene 1 (*Wt1*) are embryonic lethal with a failure of kidney and gonad formation (Kreidberg et al., 1993), the knowledge of the role of the WT1 in development has steadily increased. (Hastie, 2017). It is now well documented that the importance of WT1 during embryogenesis extends far beyond the genitourinary system and also includes the mesothelium (Kreidberg et al., 1993; Moore et al., 1999), spleen (Herzer et al., 1999), adrenal gland (Moore et al., 1999), hematopoietic (Alberta et al., 2003) and nervous system. (Wagner et al., 2002; Wagner et al., 2005). The *WT1* gene encodes a zinc finger protein with more than 30 mammalian isoforms resulting from the use of variant transcriptional and translational start sites, alternative pre-mRNA splicing and RNA editing. (Hastie, 2017). Non-mammalian vertebrates have only two WT1 isoforms, which differ by the insertion/exclusion of three amino acids, lysine, threonine and serine (KTS), between zinc fingers three and four. (Haber et al., 1991). WT1 (-KTS) molecules without the KTS tripeptide insertion function as transcription factors, and much knowledge about the role of WT1 in development and disease has been obtained from the identification of downstream target genes. (Toska and Roberts, 2014; Hastie, 2017). By interacting with other protein binding partners, WT1 is converted from an activator to a repressor of transcription. (Carpenter et al., 2004; Toska and Roberts, 2014). Compared to the WT1 (-KTS) variants, WT1 isoforms harboring the tripeptide insertion in their zinc finger domain have a higher RNA binding affinity and may operate predominantly through post-transcriptional mechanisms. (Larsson et al., 1995; Niksic et al., 2004; Bor et al., 2006). In addition, WT1 has been shown to regulate chromatin switching between an active and repressed configuration at the *Wnt4* locus. (Essafi et al., 2011). Recent findings indicate that WT1 is also involved in the epigenetic control of gene expression. (Rampal et al., 2014; Wang et al., 2015). A unifying concept of the biological processes that are regulated by WT1 in different tissues has not evolved yet. However, insights gained from *Wt1* knockout mice and target gene identification suggest that WT1 controls the reciprocal switch between a mesenchymal and epithelial cellular state. (Hohenstein and Hastie, 2006). In tissues undergoing epithelial differentiation during development, e.g. kidneys and gonads, WT1 promotes mesenchymal-to-epithelial transition (MET). (Davies et al., 2004; Essafi et al., 2011). In other tissues, such as embryonic heart and diaphragm, WT1 is necessary for the reverse process, i.e. epithelial-to-mesenchymal transition (EMT). (Martínez-Estrada et al., 2010; Hastie, 2017). This mini-review is focused on the role of WT1 in the development and maintenance of adipose tissue.

The main function of visceral (intra-abdominal) and subcutaneous white adipose tissue (WAT) is energy storage in the form of triglycerides. Interscapular brown adipose tissue (BAT) powers energy expenditure by non-shivering thermogenesis. The latter process requires uncoupling protein-1 (UCP1), a pore-forming molecule in the inner mitochondrial membrane of brown adipocytes that dissociates H^+^-fluxes from ATP synthesis. (Chouchani et al., 2019; Ikeda and Yamada, 2020; Bertholet and Kirichok, 2021). Marked differences exist between visceral and subcutaneous WAT. While intra-abdominal obesity correlates with increased mortality, subcutaneous WAT is considered as being protective. (Wajchenberg, 2000; Pischon et al., 2008; Manolopoulos et al., 2010). Unlike subcutaneous fat deposition, visceral obesity is associated with chronic diseases including type 2 diabetes, atherosclerosis and cancer. (Stefan, 2020). Intra-abdominal fat accumulation also has a chronic inflammatory component with elevated serum levels of cytokines, which may contribute to impaired metabolism in obesity. (Silveira Rossi et al., 2021). It is still a matter of debate whether adipose tissue inflammation is cause or consequence of insulin resistance. (Burhans et al., 2018; Shimobayashi et al., 2018). In general, subcutaneous WAT is more sensitive to insulin than visceral WAT, and intra-abdominal obesity correlates with insulin resistance. (Longo et al., 2019). Several conditions may account for the detrimental effect of intra-abdominal fat accumulation. The “portal vein theory” proposes that free fatty acids and cytokines released from visceral WAT are directly transported to the liver, where they might cause organ damage. This hypothesis is supported by data showing that transplanted epididymal fat pads cause impaired glucose tolerance and hepatic insulin resistance in recipient mice only when they drain into the portal ciruculation. (Rytka et al., 2011). Due to its anatomical localization, visceral WAT is exposed to potentially harmful gut microbiota-derived products. (Geurts et al., 2014; Hersoug et al., 2016). Among those, reabsorption of bacterial lipopolysaccharides (LPS) across the intestinal mucosa and subsequent uptake from the circulation by adipocytes can promote a local inflammatory response. (Hersoug et al., 2016). Furthermore, intrinsic differences may exist between subcutaneous and visceral WAT depots. This view is supported by transplantation experiments demonstrating that subcutaneous but not visceral adipose tissue reduces body weight, blood glucose and insulin levels in grafted mice. (Tran et al., 2008). Moreover, single-cell RNA sequencing identified a class of adipocyte progenitors that are unique to visceral adipose tissue (Vijay et al., 2020), and developmental and functional heterogeneities of (pre)adipocytes may exist even within a single WAT depot. (Hwang and Kim, 2019; Lee et al., 2019; Vishvanath and Gupta, 2019).

Visceral and subcutaneous WAT also differ in their browning capacity. Browning describes the phenomenon that classical thermogenic genes, e.g. *UCP1, PRDM16* and *PPARGC1A*, are switched-on in WAT upon exposure to appropriate stimuli such as prolonged cold exposure and treatment with *β*3-adrenergic agonists. (Herz and Kiefer, 2019; Moreno-Navarrete and Fernandez-Real, 2019; Van Nguyen et al., 2020; Mu et al., 2021). It is currently unclear whether WAT browning is due to the recruitment of beige/brite (brown-in-white) adipocytes capable of thermogenic gene expression from a distinct population of progenitor cells and/or the interconversion of mature white to beige adipocytes. (Barbatelli et al., 2010; Lee et al., 2012). Ultimately, which one of the two routes is taken to generate beige adipocytes in WAT may depend on multiple conditions including the intensity of the underlying stimulus, environmental factors, the genetic background and epigenetic mechanisms. (Herz and Kiefer, 2019) The browning capacity varies considerably between different WAT depots. Thermogenic genes in mice can be induced more readily in subcutaneous than visceral WAT. (Vitali et al., 2012; Jia et al., 2016) While it is still a matter of controversy whether this is valid also for humans (Sidossis et al., 2015; Zuriaga et al., 2017), WAT browning has gained considerable interest for its potential use to tackle obesity and metabolic disease. (Cheng et al., 2021; Wang and Wei, 2021).

### WT1 is Required for the Development and Maintenance of Visceral Fat Depots

Chau *et al.* were the first showing that the physiological significance of WT1 is not restricted to embryogenesis but spans the entire lifetime. (Chau et al., 2011). They used a tamoxifen-inducible transgenic approach to demonstrate that ubiquitous *Wt1* deletion in mature mice causes acute multiple organ failure including glomerular kidney injury, atrophy of the exocrine pancreas and impaired erythropoiesis. (Chau et al., 2011). Surprisingly, WT1-depleted adult mice also exhibit a strongly reduced bone and fat mass, both tissues sharing their origin from common mesenchymal stem cells. (Chau et al., 2011; Favaretto et al., 2021). Fat loss in mice with induced *Wt1* knockout affects both, the intra-abdominal WAT and the interscapular BAT. (Chau et al., 2011). A recent study confirmed the reduction of mesenteric fat in WT1 depleted adult mice. (Wilm et al., 2021). Lineage tracing experiments in mice endorse the contribution of mesothelium-derived *Wt1* expressing cells to all visceral WAT depots. (Chau et al., 2014). *Wt1* expressing cells do not contribute to the BAT lineage, which originates from Myf5^+^ cells in the paraxial mesoderm nor to subcutaneous WAT, whose developmental origin is not well understood. (Chau et al., 2014). Hence, the reduction of BAT mass in adult mice with induced *Wt1* deletion does not reflect a cell autonomous defect of brown (pre)adipocytes, but is possibly related to the suppressed IGF-1 serum levels of these animals. (Chau et al., 2011). This assumption, which has not been proven yet, is supported by the atrophy of BAT following conditional deletion of the IGF-1 receptor in mouse adipose tissues (Boucher et al., 2016).

In all visceral WAT depots, WT1 can be detected in the stromal vascular fraction (SVF) containing the fat cell progenitors (preadipocytes), endothelial and immune cells. *Wt1* is not expressed in mature adipocytes. (Chau et al., 2014). Importantly, the contribution of *Wt1* expressing cells to visceral WAT is not terminated at the end of gestation, but a subset of fat appendages, particularly in the epididymal region, continue to arise from WT1-positive progenitor cells postnatally. (Chau et al., 2014). It is tempting to speculate, whether WT1 determines the fate of a subpopulation of progenitor cells in visceral WAT. (Chau and Hastie, 2015). This idea is corroborated by the observation that adipocytes derived from WT1-positve *vs.* WT1-negative progenitor cells differ in their size and lipid droplet distribution. (Chau et al., 2014). Furthermore, microarray hybridization experiments identify WT1 as one out of only three transcription factors showing a visceral fat-selective expression profile in mice. (Cohen et al., 2014).

WT1-positive SVF cells can be induced to differentiate *in vitro* not only to adipocytes but also to muscle cells and–to a lesser extent–osteoblasts. (Chau et al., 2014). The osteoblast forming capacity differs among visceral WAT depots suggesting functional heterogeneity even within the subpopulation of WT1-positive prognitors. (Chau et al., 2014) A recent lineage tracing study by Wilm *et al.* shows that *Wt1* expressing mesothelial cells in adult peritoneum do not contribute to the deeper stromal and parenchymal compartments in the abdominal cavity, but rather constitute the progenitor niche for visceral WAT. (Wilm et al., 2021) It is well documented that adult mesothelial cells can undergo epithelial-to-mesenchymal transition (EMT) under challenging conditions such as peritoneal injury. (Han et al., 2019; Lho et al., 2021) Considering the established role of WT1 in EMT (Martínez-Estrada et al., 2010; Essafi et al., 2011; Hastie, 2017), one can hypothesize whether WT1 enables progenitor cells in the mesothelium to acquire a mesenchymal phenotype and provide a pool of adipocyte progenitors. However, the developmental origin of visceral fat from mesothelium has been challenged by a recent study. Using single-cell RNA sequencing, Westcott *et al.* show that WT1 is not restricted to visceral adipose mesothelium but also expressed in a population of *Pdgfra* ^*+*^ and *Sca-1* ^*+*^ preadipocytes in mice and humans. (Westcott et al., 2021). These authors identify keratin 19 (Krt19) as a highly specific marker for adult mouse mesothelium and demonstrate that Krt19^+^ cells do not differentiate to adipocytes *in vitro*, nor do they contribute to the pool of adipocytes in visceral fat depots *in vivo*. (Westcott et al., 2021). Furthermore, studies incorporating single-cell RNA sequencing in murine visceral WAT detected *Wt1* in non-mesothelial stromal cell polulations. (Burl et al., 2018; Hepler et al., 2018). According to these data, *Wt1* expressing preadipocytes are distinct from *Wt1* expressing mesothelial cells.

### WT1 Represses Thermogenic Genes

Recent studies demonstrate that WT1 represses a classical BAT genetic signature in visceral WAT. Thus, SVF cells isolated from visceral WAT of transgenic mice with adipocyte-specific deletion of *Wt1* express thermogenic genes including *Ucp1*, *Prdm16* and *Cidea*. (Cohen et al., 2014) PRDM16 is a transcriptional co-regulator that controls the developmental switch between skeletal muscle myoblasts and brown adipocytes from common Myf5^+^ progenitors. (Seale et al., 2008). *Prdm16* is highly expressed in the interscapular BAT and significantly elevated in subcutaneous compared to visceral fat depots. (Cohen et al., 2014). Adipocyte-selective deletion of the *Prdm16* gene in mice abrogates thermogenic gene expression in beige adipocytes, while leaving the function of classical BAT intact. (Cohen et al., 2014). When kept on high-fat diet, mice lacking PRDM16 in their adipocytes acquire a phenotype of visceral obesity with insulin resistance, hepatic steatosis and subcutaneous macrophage infiltration. (Cohen et al., 2014). Conversely, transgenic overexpression of *Prdm16* driven by the aP2-promoter in adipose tissues gives rise to beige adipocytes in subcutaneous but not in epididymal WAT. (Seale et al., 2011). Hence, it is unlikely that the lower levels of endogenous PRDM16 account for the poorer browning susceptibility of visceral *vs.* subcutaneous WAT. Instead, yet unknown factors may exist in visceral WAT that confer resistance to browning stimuli, and WT1 might be one of those. The successive decline of *Prdm16* transcripts in differentiating primary preadipocytes was associated with increasing levels of *Wt1* mRNA suggesting that the browning inducer PRDM16 and WT1 are reciprocally regulated. (Cohen et al., 2014). It would be worthwhile to investigate in future studies whether PRDM16 functions as a direct inhibitor of *Wt1* expression during adipocyte differentiation.

**FIGURE 1:**
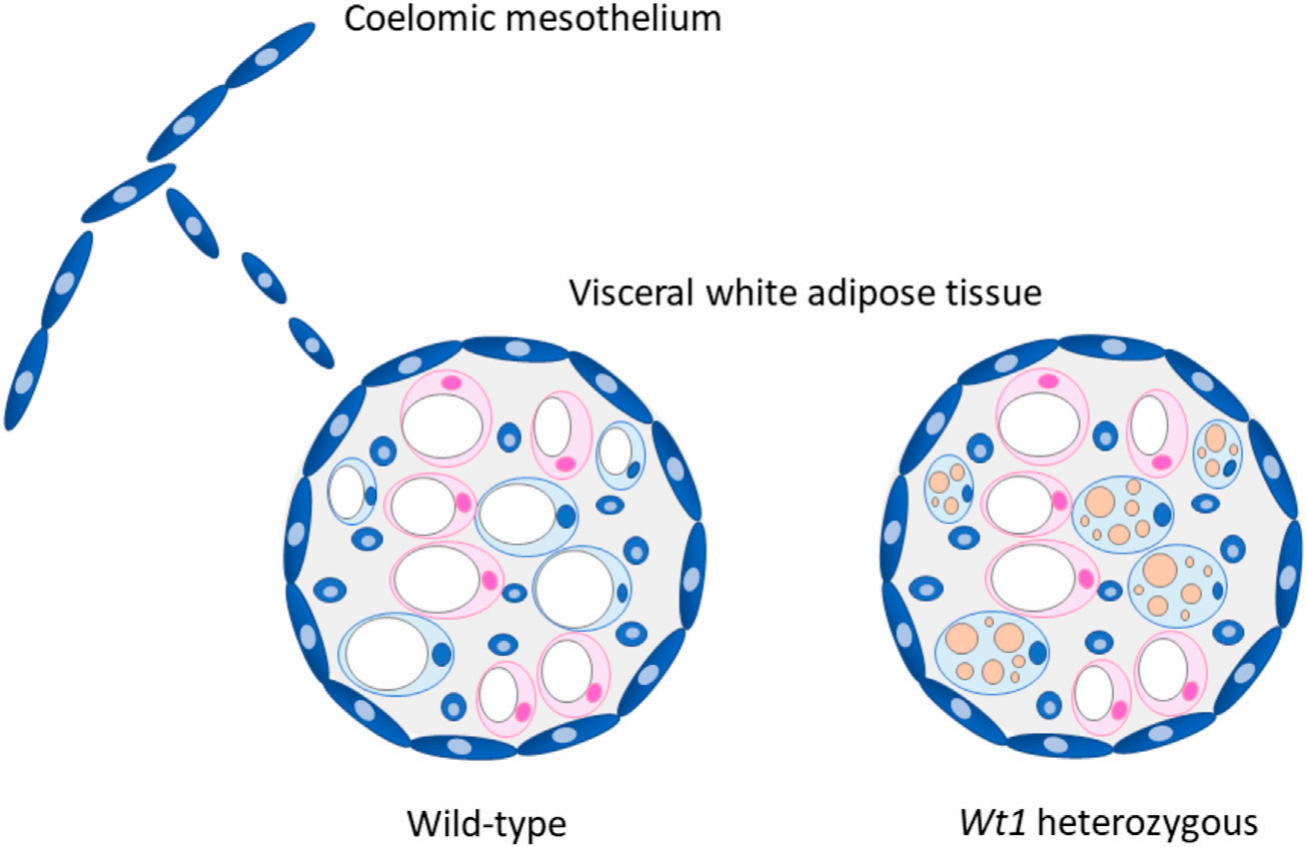
Proposed role of WT1 in visceral white adipose tissue (WAT). WT1 expressing cells (blue) derived from the coelomic mesothelium possibly contribute to the visceral fat depots, which are covered with a mesothelial cell layer. (Chau et al., 2014). Heterozygous *Wt1* knockout mice show morphological and molecular signs of browning in their visceral (epididymal) WAT. (Kirschner et al., 2022). It is currently unknown whether epididymal WAT browning is restricted to adipocytes originating from WT1 expressing progenitor cells (blue) as drawn in the figure, or also includes WT1-negative cells (pink). The marked multilocular fat deposition in beige adipocytes is drawn for the sake of clarity but not seen in heterozygous *Wt1* knockout mice. (Kirschner et al., 2022). Adapted with modifications from ref. (Chau and Hastie, 2015).

In the light of the above, we reasoned that WT1 might prevent a thermogenic gene expression program in visceral WAT. We addressed this issue by combining *in vitro* differentiation of brown preadipocytes with *in vivo* analyses of WAT depots in wild-type and heterozygous *Wt1* knockout mice. Retroviral delivery of WT1 repressed thermogenic genes upon *in vitro* differentiation of immortalized brown preadipocytes. (Kirschner et al., 2022). Likewise, overexpression of *Wt1* reduced *Ucp1, Ppargc1a, Cidea, Prdm16* and *Cpt1b* transcripts in differentiating Sca1^+^:CD45^−^:CD31^−^ preadipocytes isolated from the interscapular BAT of mice*.* (Kirschner et al., 2022). WT1 caused no changes of adipocyte-selective genes that are expressed in both, white and brown fat cells. WT1 also did not interfere with overall adipogenic differentiation assessed in terms of intracellular lipid storage. (Kirschner et al., 2022). These findings let us conclude that ectopic WT1 suppresses the genetic signature of brown adipocytes. This idea is strengthened by the observation that adipocytes arising from WT1-positive progenitors in epididymal WAT contain fewer but larger lipid droplets in their cytoplasm (Figure 1). (Chau et al., 2014)

**FIGURE 2:**
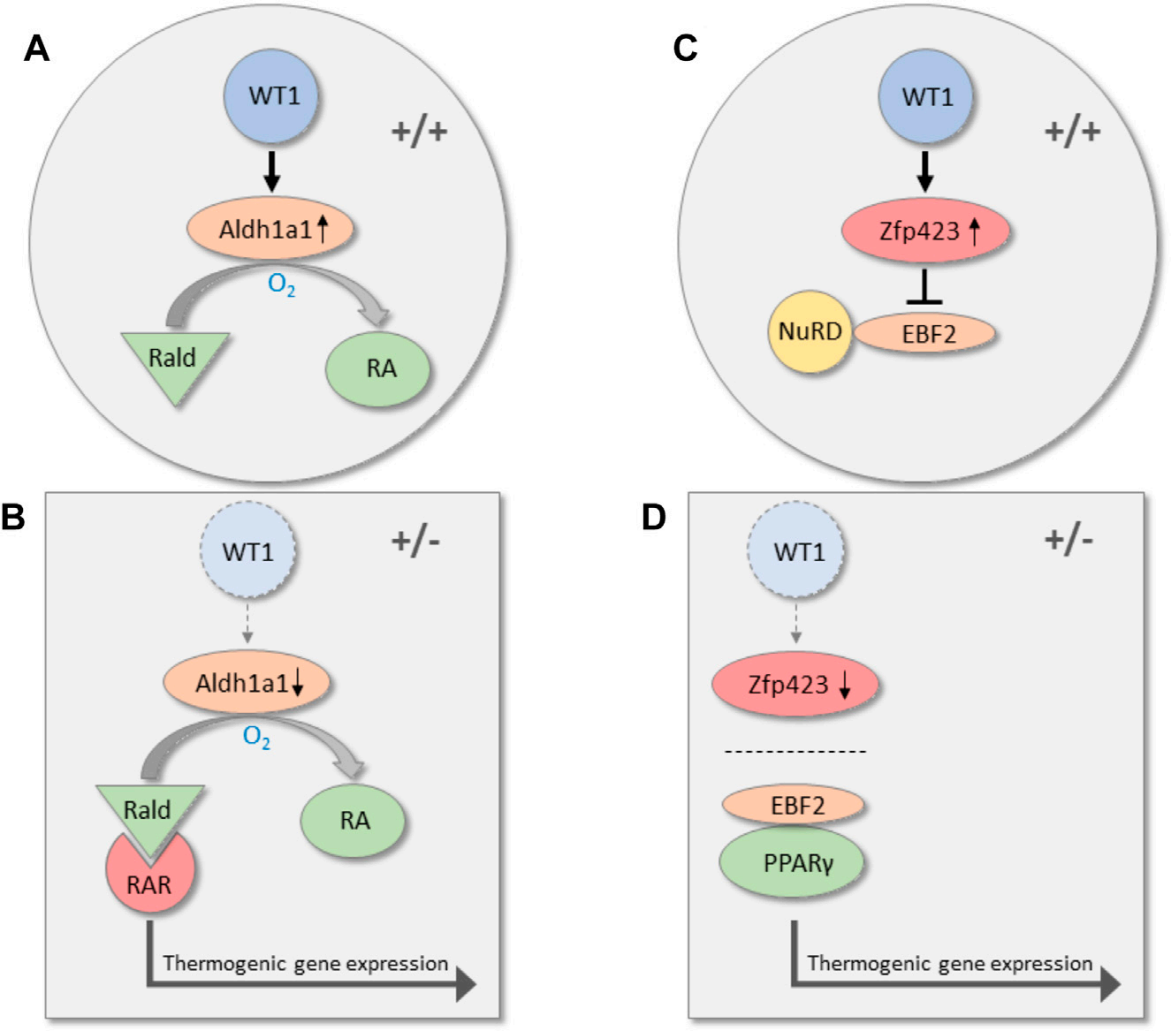
Transcriptional pathways along which WT1 may repress thermogenic genes in visceral WAT. In wild-type mice (+/+), WT1 increases the expression of Aldh1a1, which catalyzes the oxidation of retinaldehyde (Rald) to retinoic acid (RA) **(A)**. Reduction of Aldh1a1 in visceral WAT of heterozygous *Wt1* knockout mice (+/−) causes accumulation of retinaldehyde (Rald), which stimulates thermogenic gene expression via retinoic acid receptor (RAR) activation **(B)**. (Kiefer et al., 2012) WT1 is also required for normal expression of the transcription factor Zfp423 in visceral WAT. Zfp423 recruits the NuRD co-repressor complex and thereby prevents the transcription factor EBF2 from activating thermogenic genes **(C)**. (Shao et al., 2021). Zfp423 is reduced in visceral WAT of mice with a single *Wt1* allele (+/−). (Kirschner et al., 2022). As a consequence, interaction of PPAR*γ* with EBF2 shifts the occupancy to thermogenic gene promoters and induces thermogenic gene expression **(D)**.

To identify potential WT1 target genes, we silenced endogenous *Wt1* in murine epididymal SVF cells by RNA interference. Knockdown of *Wt1* reduced *Aldh1a1* and *Zfp423* transcripts in these cells. On the other hand, both RNAs increased significantly upon forced expression of WT1 in brown preadipoytes. (Kirschner et al., 2022). Targeted inactivation of *Aldh1a1* and *Zfp423* has been reported to induce thermogenic genes in WAT of mice. (Kiefer et al., 2012; Shao et al., 2016; Hepler et al., 2017). ALDH1A1 catalyzes the oxidation of retinaldehyde (Rald) to retinoic acid, and *Aldh1a1* deficiency causes accumulation of Rald in WAT. (Ziouzenkova et al., 2007; Molotkov and Duester, 2003). Rald stimulates the expression of *Ucp1* and other thermogenic genes in white fat cells by activating the retinoic acid receptor (RAR) (Figure 2). (Kiefer et al., 2012) The transcription factor ZFP423 represses thermogenic genes by inhibiting the activity of the transcriptional co-regulator EBF2. Mechanistically, ZFP423 recruits the NuRD co-repressor complex that prevents EBF2 from activating thermogenic genes (Figure 2). (Shao et al., 2021) Disruption of the ZFP423-EBF2 protein interaction induces a shift in PPARγ occupancy of thermogenic genes and elicits widespread WAT browning in adult mice (Figure 2). (Shao et al., 2016; Shao et al., 2021) ChIP-sequencing analysis of genomic WT1 binding sites in mouse embryonic kidneys classify *Zfp423* among the top 1,000 genes (*p*-value 4.7 × 10^–23^). (Motamedi et al., 2014). These data suggest that WT1 stimulates *Aldh1a1* and *Zfp423* expression in epididymal SVF cells either directly or through indirect mechanisms. By increasing ALDH1 and ZFP423 levels, WT1 presumably represses a genetic program of classical BAT in white preadipocytes. Notably, inactivation of *Aldh1a1* and *Zfp423* in mice causes WAT browning not only in intra-abdominal but also in subcutaneous fat depots indicating that these molecules do not convey a specific action of WT1 in visceral WAT. (Kiefer et al., 2012; Shao et al., 2016; Shao et al., 2021). Genome-wide approaches combining RNA deep sequencing with ChIP sequencing technology might give a more complete picture of the transcriptional events that are regulated by WT1 in visceral fat cell progenitors.

### Heterozygous *Wt1* Knockout Mice Show Improved Glucose and Lipid Metabolism

Using mice with a heterozygous *Wt1* gene, we next examined whether WT1 inhibits thermogenic gene expression also in visceral WAT *in vivo*. Unlike the embryonic lethal full knockout, *Wt1* heterozygous mice are viable and lack obvious developmental abnormalities. (Kreidberg et al., 1993). Strikingly, *Wt1* heterozygosity is associated with molecular and morphological signs of browning including elevated UCP1 levels in epididymal WAT (Figure 1). (Kirschner et al., 2022) No differences in thermogenic gene expression in interscapular BAT and subcutaneous WAT, i.e. in WT1-negative fat depots, are detectable between wild-type and heterozygous *Wt1* knockout mice. (Kirschner et al., 2022). These findings suggest that WT1 is necessary for maintaining a white adipose identity in epididymal WAT. Notably, *β*3-adrenergic stimulation increases thermogenic gene expression to a similar extent in wild-type and heterozygous *Wt1* knockout mice suggesting that WT1 does not limit the overall browning capacity of epididymal WAT. (Kirschner et al., 2022).

Inhibition of beige fat cell function by adipocyte-specific deletion of *Prdm16* in mice causes severe metabolic disorder with insulin resistance and diet-induced fatty liver disease. (Cohen et al., 2014). This observation prompted us to examine whether differences in glucose and lipid metabolism exist between wild-type and heterozygous *Wt1* knockout mice. Compared with their wild-type littermates, *Wt1* mutant mice exhibit significantly improved whole-body glucose tolerance and much weaker hepatic steatosis when kept on a high-fat diet for 11 weeks. (Kirschner et al., 2022). Superior metabolic function is observed also in other genetic mouse models of enhanced WAT browning including deletion of *Ago1* in vascular endothelial cells (Tang et al., 2020), ROCK2 depletion (Wei et al., 2020), and adipose-specific knockout of *Hoxc10*. (Tan et al., 2021). We therefore assume that improved metabolic health of heterozygous *Wt1* knockout mice is related to the activation of a thermogenic program in their visceral WAT. However, other cell types and tissues might be involved as well. Notably, *Wt1* expressing cells delaminating from the coelomic epithelium contribute to the pool of stellate cell progenitors in mouse liver. (Ijpenberg et al., 2007). Hepatic stellate cells are the major storage site of retinyl esters in the body. (Haaker et al., 2020). Following hepatic injury, retinol is released from these cells and partially converted to retinoic acid (RA) by the enzymatic activity of retinaldehyde dehydrogenases (RALDH). (Haaker et al., 2020). Interestingly, *Aldh1a2*, the predominant isoform that encodes RALDH2 in embryonic tissues, is a direct downstream target gene of WT1 in developing epicardial cells. (Guadix et al., 2011). Retinoid signaling is important for normal liver function, and serum levels of retinol and RA are reduced in non-alcoholic fatty liver disease. (Saeed et al., 2017). Mice heterozygous for *Rdh10*, a gene encoding retinol dehydrogenase, develop glucose intolerance and severe hepatic steatosis under high-fat diet. Their phenotype can be rescued by treatment with all*-trans* RA. (Yang et al., 2019). These data raise the possibility that impaired retinoid signaling of hepatic stellate cells contributes to the metabolic abnormalities of heterozygous *Wt1* knockout mice. The generation of transgenic mouse lines with conditional *Wt1* deletion in progenitor cells of visceral WAT and hepatic stellate cells can shed some light onto this issue.

## Conclusion, Perspectives and Open Questions

Visceral WAT is a prime example for the complex role of WT1 reaching from embryogenesis to adulthood. Recent data suggest that WT1 is necessary for maintaining white adipose identity in visceral WAT. (Kirschner et al., 2022). Further studies including genome-wide single cell sequencing technologies may help to identify WT1 downstream target genes and elucidate the molecular mechanisms by which WT1 represses browning processes in visceral WAT. Strikingly, glucose and fat metabolism are better preserved in *Wt1* heterozygous than in wild-type mice under high-fat diet. (Kirschner et al., 2022). Circumstantial evidence suggests that improved metabolic function of heterozygous *Wt1* knockout mice is due to the expression of *Ucp1* and other thermogenic genes in their visceral WAT, a phenomenon referred to as browning. This hypothesis needs to be proven by generating and characterizing mouse lines with selective deletion of *Wt1* in white preadipocytes. These transgenic mice might also be useful for identifying WT1-dependent transcriptional networks in fat cell development. Another important issue is to clarify whether WT1 determines the white adipocyte fate also in visceral WAT in humans. If so, this could fuel further studies aiming to establish WT1 as a potential therapeutic target in metabolic disorders.
